# Addressing Motor Dysfunction by a Selective α6-Containing Nicotinic Receptor Antagonist

**DOI:** 10.1021/acs.jmedchem.3c01817

**Published:** 2023-10-18

**Authors:** Cristiano Bolchi, Marco Pallavicini

**Affiliations:** Dipartimento di Scienze Farmaceutiche, Università degli Studi di Milano, via Mangiagalli 25, I-20133 Milano, Italy

## Abstract

In the striatum, presynaptic α6-containig nicotinic receptors are crucially involved in the modulation of dopamine release. CVN417, a novel selective antagonist at this receptor subtype, attenuates motor dysfunction in a Parkinson’s disease-relevant animal model, suggesting, for this pathology, a therapeutic strategy that could greatly profit from the restricted localization of α6* nicotinic receptors in the brain.

Parkinson’s disease (PD) is classified among the 18 major neurological disorders globally, with its rank of disease burden moved up from 14th in 1990 to 10th in 2019.^1^ Dysregulation of basal ganglia (BG) circuitry is crucially implicated in this neurodegenerative pathology, whose prominent symptoms are motor dysfunctions, resting tremors, and postural instability. Within BG circuitry, the striatum is the major processing unit. About 95% of striatal neurons are GABAergic projections neurons, named medium spiny neurons (MSNs). The remaining ∼5% of striatal neurons are about half-and-half GABAergic and cholinergic interneurons (ChIs) (Figure 1).^2^

**Figure 1:**
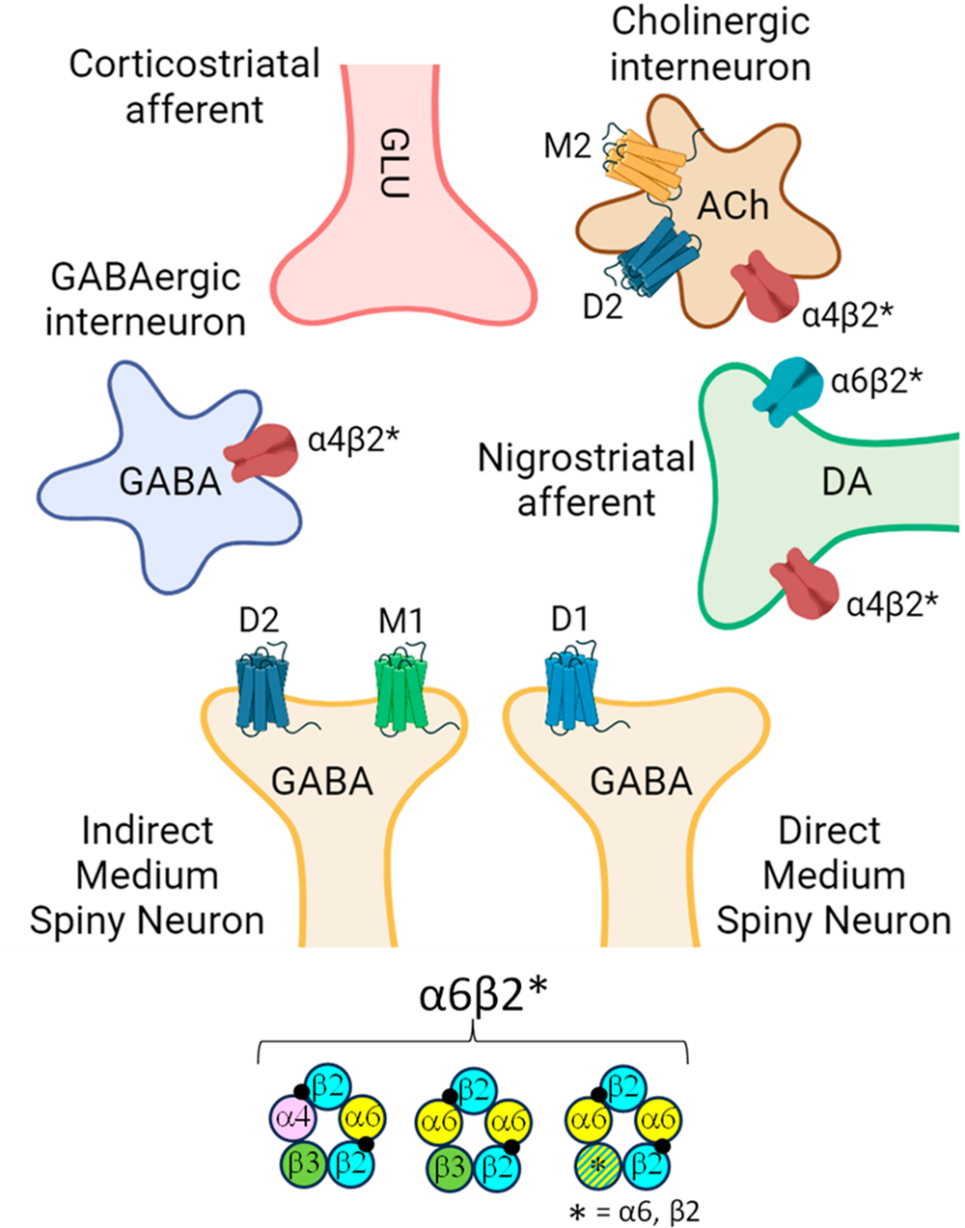
Simplified illustration of the different types of striatal neurons. For clarity, only the receptor subtype localizations discussed in the text are shown (D_1_, D_2_: dopamine receptors; M_1_, M_2_: muscarinic acetylcholine receptors; α4β2*, α6β2*: nicotinic acetylcholine receptors). Abbreviations: ACh, acetylcholine; DA, dopamine; GABA, γ-aminobutyric acid; GLU, glutamate. The closed black circles represent the agonist binding sites.

The activity of the striatum is mainly driven, in addition to excitatory glutamatergic afferents from the cortex, by modulatory nigrostriatal dopaminergic afferents, where presynaptic nicotinic acetylcholine receptors containing at least one α6 subunit (α6β2*-nAChRs; the asterisk denotes the possible presence of additional subunits) are selectively localized (Figure 1).^2^ In a recent issue of the *Journal of Medicinal Chemistry*, the Bürli group at the Cambridge drug discovery company Cerevance reported a novel brain-penetrant α6-containing nicotinic receptor antagonist, CVN417 (Figure 2), that modulates phasic dopaminergic neurotransmission in mouse striatum and attenuates tremors in a PD-relevant animal model.^3^

**Figure 2:**
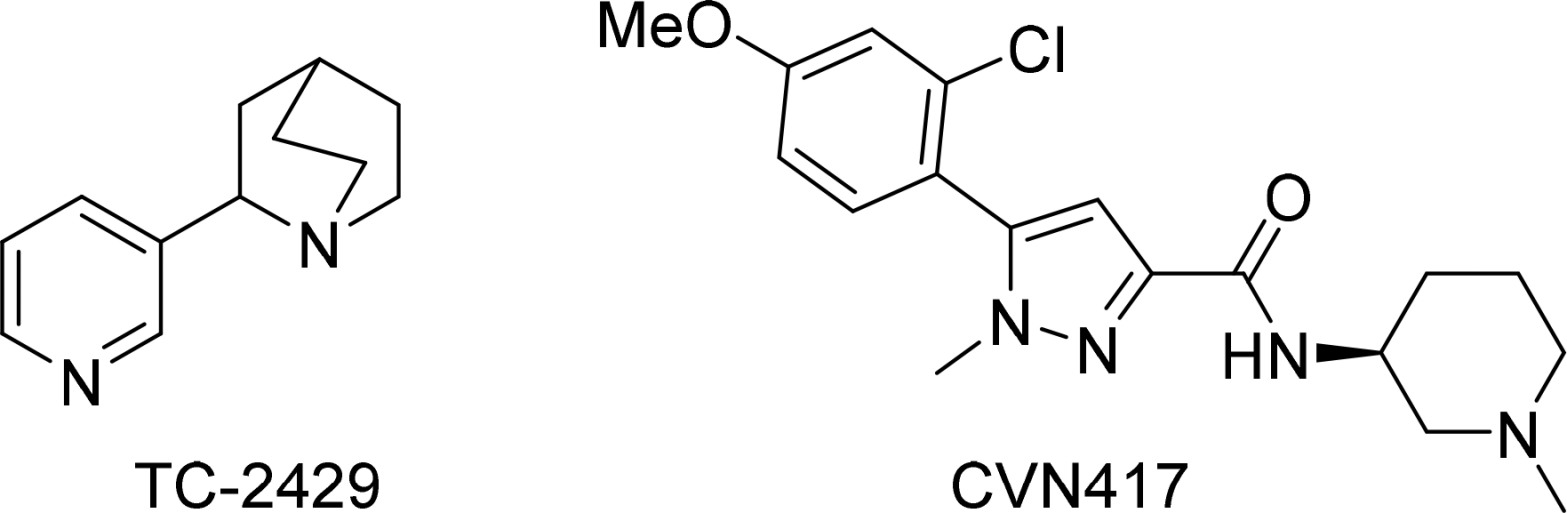
Structures of TC-2429 and CVN417, the full agonist and antagonist, respectively, at the α6-containing nicotinic receptor.

Modulatory dopaminergic afferents act through two main pathways, referred to as the direct pathway, mediated by excitatory D_1_ receptors (D_1_Rs) and promoting movement, and the indirect pathway, terminating ongoing movement and predominantly influenced by inhibitory D_2_ receptors (D_2_Rs).^2^ A striatal dopamine (DA) deficit is classically thought to underlie many of the movements disorders in PD because it results in reduced activation of the direct pathway and reduced inactivation of the indirect pathway.^2^l-DOPA, the first drug of choice when treating PD, is used to counteract dopamine deficiency inasmuch as it is the dopamine precursor capable of crossing the blood–brain barrier.

Imbalanced direct and indirect pathway signaling is accentuated by elevated striatal ACh, resulting from reduced activation of D_2_Rs on striatal ChIs, which activates the indirect pathways via M_1_Rs on MSNs. ChIs are a small fraction of all striatal neurons, but they have a great influence over striatal motor outputs, and they express a multitude of receptor subtypes, which could allow ACh transmission to be modulated, though not without unwanted side effects. Therefore, normalizing ChI overactivity remains, to date, a pursuable goal for the treatment of PD at disease stages when ChIs are still intact.^2^

Targeting muscarinic receptors is an alternative therapeutic option. Stimulating M_2_Rs on ChIs lowers ACh levels, while blocking M_1_Rs on MSNs attenuates the indirect pathway. However, even these approaches are not without drawbacks and adverse effects.^4^

Within this context, striatal nicotinic ACh receptors (nAChRs) can play an untapped distinctive role as viable targets to improve parkinsonian state. Notably absent, unlike M and D receptors, from MSNs, striatal nAChRs result from different combinations of α4, α6, α7, β2, and β3 subunits, with a predominance of α6β2- and α4β2-containing receptors (α4β2*- and α6β2*-nAChRs).^2,4^ Their prevalent localization is on DAergic terminals, where α4β2*- and α6β2*-nAChRs are expressed and seem to act by depolarizing the terminal bouton and producing dopamine release.^2,5^ In contrast to the α4β2* subtype, which is widespread throughout the brain, the α6β2* subtype has a relatively selective localization to the nigrostriatal pathway, restricted, moreover, in the striatum to DAergic terminals. Such a unique distribution makes the α6β2*-nAChR, more than the α4β2* subtype, an outstanding target to treat disorders linked to the nigrostriatal system.^4^

The characterization of α6-containing nAChRs, long hampered by difficulties in expression and identification, is relatively recent. It greatly benefited from the use of α-conotoxin MII, a toxin identified in the early 2000s with high and selective affinity for these receptor subtypes.^6^ However, although a number of successive studies highlighted the importance of the striatal localization of α6β2*-nAChRs and their peculiar involvement in motor behaviors, the results have remained elusive. First of all, non-decisive or conflicting evidence about reduction of PD incidence and alleviation of PD motor symptoms arises from the experiments with both chronic and acute dosing of nicotine, a non-selective nAChR agonist directly effecting on the midbrain dopamine.^2^ Second, beneficial effects of nicotinic agonists targeting both α4β2*- and α6β2*-nAChRs are documented in L-DOPA-induced dyskinesia but not in PD models.^2,7^ Third, administration of a moderately α6β2*-selective agonist, such as TC-2429 (Figure 2), is reported to stimulate both dopamine release and locomotor activity in vivo, and its use to improve parkinsonian state is only prospected.^8^ Until recent years, the unavailability of ligands that are highly selective for α6β2*-nAChRs and suitable for testing on PD models has, in fact, made it difficult to obtain conclusive information on the druggability of this nAChR subtype, which remains, to date, only a promising potential target for treatment of PD.

This notwithstanding, in the past two decades, accumulating evidence on the importance of presynaptic location of α4β2*- and α6β2*-nAChRs has prompted a series of studies highlighting the nature and the impact of the modulation of dopamine release by acetylcholine, nicotine, and nicotinic drugs acting at these nAChRs subtypes. In a low-frequency tonic mode, dopamine neuron activity maintains low nanomolar concentrations of dopamine, while bursts of high-frequency phasic activity produce much greater release of dopamine. When dopamine neurons are quiescent (tonic firing), nAChRs are tonically active, responding to a physiologically pulsatile delivery of ACh, and their desensitization is avoided. Instead, experimentally induced high concentrations of ACh produce desensitization and decrease dopamine release at low-frequency stimulation.^7^ Nicotine, the general nicotinic antagonist mecamylamine, and the β2-selective antagonist dihydro-β-erythroidine all suppress dopamine release at tonic frequencies, whereas they increase it upon high-frequency phasic bursts.^9^ These observations indicate that both desensitization, produced by an agonist (nicotine), and antagonism (mecamylamine and dihydro-β-erythroidine) serve to discriminate tonic and phasic patterns of stimulation enhancing the sensitivity of dopamine release to burst versus non-burst stimuli and that nicotinic agonists achieve the same effect as antagonists. These processes would be mainly mediated by presynaptic α6β2*-nAChRs.

The major highlight of the study by Bürli’s group^3^ is the actualization of such a potential strategy for the treatment of PD symptoms by engaging in the development of an α6*-nAChR-selective antagonist, which should be a more accessible achievement than development of a selective agonist. The researchers succeeded by screening a library of ∼650k compounds for inhibitory activity at the α6-containing nAChR. Selection of the most promising hit was followed by a relatively streamlined hit optimization, resulting in the development of CVN417 as a lead molecule. Indeed, CVN417 combines highly potent and selective α6-containing nAChR antagonism, determined in vitro and ex vivo, with high efficacy in attenuating resting tremors in an animal PD model and, on the basis of in-depth profiling by ADME and DMPK assays, good pharmacokinetic properties. Furthermore, evidence is provided that CVN417 would exert its effects in vivo, presumably by a neurotransmission regulation involving modulation of phasic DAergic neurotransmission in an impulse-dependent manner.

This research provides a novel tactic that could be applied in PD therapy. Further studies will prove whether this is, indeed, a practicable option. Additionally, it will be interesting to study the impact of selective α6-containing nAChR antagonism also on the significantly problematic non-motor symptoms associated with PD.
